# Validation of the dosimetry of total skin irradiation techniques by Monte Carlo simulation

**DOI:** 10.1002/acm2.12921

**Published:** 2020-06-19

**Authors:** Ruiqi Li, Wenchih Tseng, Qiuwen Wu

**Affiliations:** ^1^ Department of Radiation Oncology Duke University Medical Center Durham NC USA

**Keywords:** EGSnrc, electron dosimetry, Monte Carlo simulation, total skin electron irradiation, VirtuaLinac

## Abstract

**Purpose:**

To validate the dose measurements for two total skin irradiation techniques with Monte Carlo simulation, providing more information on dose distributions, and guidance on further technique optimization.

**Methods:**

Two total skin irradiation techniques (stand‐up and lay‐down) with different setup were simulated and validated. The Monte Carlo simulation was primarily performed within the EGSnrc environment. Parameters of jaws, MLCs, and a customized copper (Cu) filter were first tuned to match the profiles and output measured at source‐to‐skin distance (SSD) of 100 cm where the secondary source is defined. The secondary source was rotated to simulate gantry rotation. VirtuaLinac, a cloud‐based Monte Carlo package, was used for Linac head simulation as a secondary validation. The following quantities were compared with measurements: for each field/direction at the treatment SSDs, the percent depth dose (PDD), the profiles at the depth of maximum, and the absolute dosimetric output; the composite dose distribution on cylindrical phantoms of 20 to 40 cm diameters.

**Results:**

Cu filter broadened the FWHM of the electron beam by 44% and degraded the mean energy by 0.7 MeV. At SSD = 100 cm, MC calculated PDDs agreed with measured data within 2%/2 mm (except for the surface voxel) and lateral profiles agreed within 3%. At the treatment SSD, profiles and output factors of individual field matched within 4%; d_max_ and R_80_ of the simulated PDDs also matched with measurement within 2 mm. When all fields were combined on the cylindrical phantom, the d_max_ shifted toward the surface. For lay‐down technique, the maximum x‐ray contamination at the central axis was (MC: 2.2; Measurement: 2.1)% and reduced to 0.2% at 40 cm off the central axis.

**Conclusions:**

The Monte Carlo results in general agree well with the measurement, which provides support in our commissioning procedure, as well as the full three‐dimensional dose distribution of the patient phantom.

## 1 INTRODUCTION

Total skin electron irradiation (TSEI or TSI) has been employed for more than 50 yr as one of the most effective treatment techniques of malignant skin diseases such as mycosis fungoides, and cutaneous lymphomas.^1^, ^2^ The clinical goal is to deliver a uniform dose to the whole skin, without damaging inner organs. According to the recommendation of AAPM Task Group No.30 (TG‐30), the field size of the composite electron beam at the patient treatment plane must be approximately 200 cm in height by 80 cm in width to encompass the largest patient. Within the rectangular field, a vertical uniformity of ±8% and a horizontal uniformity of ±4% over the central 160 by 60 cm area are desired.^1^ TG‐30 also contains explicit requirements of penetration depth and accompanying megavoltage x‐ray background dose: a penetration depth ranges from approximately 5 to 15 mm or more than 50% isodose surface encompasses most lesion; the x‐ray contamination exposed to the rest of body should be as low as reasonably achievable, with a level <1% of the electron dose at dose maximum.

Multiple techniques have been used for TSI treatment. The most commonly used technique is called Stanford six dual‐field method.^1^, ^2^, ^3^ In this method, the patient stands on a platform at an extended source source‐to to‐skin distance (SSD) and the platform is rotated successively every 60 degrees. Patient arms are designed in a different position at each angle for the uniform exposure of the skin on the arms and limiting the self‐shielding of the core. The radiation beams are named commonly based on the incident direction of the beam to the patient: anterior to posterior (AP), posterior to anterior (PA), right anterior oblique (RAO), left anterior oblique (LAO), right posterior oblique (RPO), and left posterior oblique (LPO). In each position, dual electron fields with approximately ±20° angled from horizontal are delivered at an extended SSD to provide a uniform dose distribution. The deflecting angle and SSD should be optimized based on individual facility room design and linac to maximize the field uniformity. Other techniques also exist that are variant of the Stanford method.^4^, ^5^ For example, the platform on which the patient stands can rotate at a constant speed while radiation is on. In addition, some facilities utilize an additional scattering filter that can degrade the energy and broaden the beam,^4^ ensuring an appropriate dose homogeneity. Hence, a small treatment room with a shorter SSD can be used to perform TSI treatment.

Although both Stanford technique and rotational technique^4^ provide a uniform dose distribution at the treatment plane, it requires that the patient remains standing for the entire treatment duration, which could be a safety issue for patients who are too weak to stand and therefore unable to endure the procedure. To tackle the problem, a TSI lay‐down technique was first proposed by Wu et al.^6^ and further modified by Deufel and Antolak,^7^, ^8^ which maintained the advantage of the Stanford technique while allowing the patient on a more comfortable lying position during the treatment. In this technique, AP/PA fields were delivered with the patient’s umbilicus positioned directly under the Linac head; LAO/LPO/RAO/RPO fields were delivered at a 60° gantry angle with junction fields. Furthermore, Deufel and Antolak^7^ proposed a novel hybrid method, in which a customized filter was designed to broaden the electron beam to compensate for the reduced SSD (target to floor distance is usually <230 cm). As a result of the addition of the filter, a single beam can be used for oblique fields to satisfy the dose uniformity requirement, eliminating the need for field junctioning, hence the setup time was reduced and treatment efficiency can be significantly improved.

Currently, there is no commercial treatment planning system for TSI. The commissioning of the TSI is specific to each linac room and a comprehensive list of dosimetric measurements is required. The treatment planning is largely based on the measurement data from the commissioning process and doses at a few representative points over the skin are calculated and measured for each patient.

Monte Carlo (MC) simulation is a sophisticated method in dose estimation and has been gradually accepted as an alternative dose calculation in the radiotherapy field.^9^ Furthermore, it can provide much more than just the doses at a limited number of points, such as the volumetric dose distribution, making it an ideal tool to validate the commissioning results, and to provide guidance for further optimization of the treatment technique.

Due to the limited number of facilities performing the TSI, few MC studies have been published to simulate the dosimetric effects of TSI: Pavon et al.^10^ used EGS4 code^11^ to estimate the dosimetric properties of their TSI technique; Ye et al.^12^ used the MC method to estimate the dosimetry of the beam penetrating a specific beam modifier that can be applied in TSI techniques; Nevelsky^13^ validated the measured dosimetry of their own dual‐field TSI technique by MC simulation in a CT‐based anthropomorphic phantom, they also studied the room scatter effect of the TSI by the MC method.^14^

Previous TSI MC studies were mainly focused on the Stanford technique, in which only one unique field needs to be simulated and the composite is the summation of the transformation of that single field. On the contrary, the lay‐down technique was more complex, with two different SSDS and three unique fields to be simulated. In this study, we presented a MC framework to simulate the TSI treatment, including both stand‐up and lay‐down techniques. Dosimetric data acquired during the commissioning process were used to validate the MC simulation. Additional data such as full three‐dimensional (3D_dose distributions were also presented.

## 2 MATERIALS AND METHODS

In this section, we first describe the beam characterization and the setup for TSI stand‐up and lay‐down techniques currently implemented. Then we present in detail the MC models that we developed for simulating TSI techniques under EGSnrc and VirtuaLinac (VL) environment. Finally, we introduce the analysis and comparison metrics between MC simulation and measurement.

### 2.A Description of TSI techniques

#### 2.A.1 TSI stand‐up technique

Treatment was performed on a Varian TrueBeam Stx Linac in the 6 MeV HDTSe mode with a dose rate of 2500 MU/min. Six pairs of electron beams were used in this technique. For each beam pair, the collimators (X and Y jaws) were set to the standard 36 × 36 cm^2^ field at isocenter and electron applicator were removed to maximize the file size. MLC was fully retracted. The beams were weighted equally with gantry angles set to 251° and 289° (tilted ±19° from 270°) for optimal dose uniformity. The patient stands on a platform that can rotate at steps of 60°, and the patient's surface is positioned 300 cm away from the source (200 cm from isocenter). To improve the treatment efficiency and reduce patient fatigue, we delivered the total six pairs of electron beams for the first and second fraction, when in vivo dose measurements were performed; then we split a single fraction into two sub‐fractions: three pairs of beams at 120° apart with twice the MUs were used on one day and the other three pairs on the other day, and they alternated at each fraction so a complete six pairs of beams are used to achieve prescribed dose after every two fractions.

#### 2.A.2 TSI lay‐down technique

Treatment was performed on the same TrueBeam Stx in 6 MeV HDTSe mode. Ten electron beams were used in this technique, based on the relative position between patient and linac. Secondary collimators (X and Y jaws) were set to 30 × 40 cm^2^ at isocenter and electron applicator was removed. A customized Cu scattering filter is placed on the interface mount: A 0.25 mm copper disk is positioned between two 1 mm polycarbonate rectangular layers, and corners of polycarbonate layers were trimmed by 5 cm.^8^

**Fig. 1:**
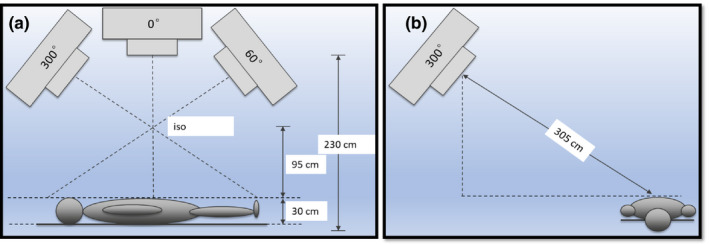
Setup of the TSI lay‐down technique, the patient is assumed of 30 cm thickness and lies on a polycarbonate platform, distance from platform to the floor is 5 cm; (a) anterior–posterior (AP)/posterior–anterior (PA) treatment fields (b) left posterior oblique (LPO), right posterior oblique (RPO), left anterior oblique (LAO), and right anterior oblique (RAO) treatment fields.

The setup of the TSI lay‐down technique is shown in Fig. 1;, the patient is modeled by a 30 cm diameter cylinder. For anterior–posterior (AP) and posterior–anterior (PA) positions, the patient’s umbilicus is positioned below the isocenter at SSD of approximately 195 cm, and the patient is oriented perpendicular to the linac waveguide so the cranial‐caudal axis is in the plane of gantry rotation. The beams from AP and PA are identical, each consists of three overlapping sub‐beams with gantry angles of 300°, 0°, and 60°, named AP_G300, AP_G0, AP_G60, and similarly PA_G300, PA_G0, and PA_G60, respectively. Each individual beam is carefully weighted to achieve a uniform dose relative profile at 1cm depth (close to d_max_). For the left anterior oblique (LAO), right anterior oblique (RAO), left posterior oblique (LPO), and right posterior oblique (RPO) field (named as oblique fields or OB field), the gantry angle is set to 300°, and the platform with the patient is pulled out by 212 cm and rotated so it is positioned parallel to the waveguide. The extended SSD is approximately 305 cm.

Treatment parameters for the two techniques are summarized in Table 1. The amount of MU in lay‐down technique is approximately twice of those in the stand‐up technique, this is partly due to the attenuation effect of the copper filter, and the use of three sub‐fields in the AP/PA direction rather than two in the stand‐up technique. This may lead to an increased risk of ozone production in the treatment room, which was found to be within acceptable limits during commissioning. However, since the patient just needs to lie on the platform and flipped for one time during the whole treatment, the overall treatment time (including the patient setup and beam delivery) stays about the same as in the stand‐up technique. In addition, beams from all six directions are delivered at each fraction in the lay‐down technique, while the majority of the stand‐up technique treats only three directions per fraction.

**Table 1:** Comparison of treatment parameters for TSI stand‐up and lay‐down technique, all parameters were based on the patient setup and MU prescription in our clinic.

	Stand‐up	Lay‐down
Total MU for prescription dose of 150 cGy per fraction	7312	14084
Number of treatment field	12 (initial 2 fractions); 6 (fractions afterwards)	10
SSD (cm)	300	AP/PA:195; Oblique:305
Degrader (Cu)	No	Yes
Treatment time per fraction (min)	12 beams: 30; 6 beams: 15	20

#### 2.A.3 Commissioning and experimental measurements

Commissioning of TSET consisted of absolute calibration, measurement of dosimetric parameters of individual fields, and verification of dose distribution of the whole treatment.

The absolute dosimetry calibration was measured with a plane‐parallel ion chamber (PTW34001 ROOS, Hicksville, NY) in a water phantom at the reference condition (36 × 36 cm^2^, d = 1.5 cm, no applicator) at the SSD of 100 cm, the ion chamber was previously cross‐calibrated against a cylindrical ion chamber (PTW TN30006, Hicksville, NY) in a 16 MeV electron beam. At the treatment plane (Stand‐up: SSD = 300 cm; Lay‐down: SSD = 195/305 cm), the plane‐parallel ion chamber was placed into the solid water layers to measure the output of individual beam, and converted into the absolute dose per MU using the ratio of dosimeter reading at treatment SSD to the reading at the reference condition.

(1) $$Output\left(\frac{\mathrm{cGy}}{\mathrm{MU}}\right)=\frac{\mathrm{Reading}}{Reading_{@ref}}\cdot Output_{@ref}\left(\frac{\mathrm{cGy}}{\mathrm{MU}}\right)$$

Relative dosimetric checks included the measurement of Percentage depth dose (PDD) curves and cross‐beam profiles. At SSD = 100 cm, depth profiles along the central axis at 100 cm SSD were measured in a water tank with a plane‐parallel ion chamber. Cross‐beam profiles at 1 cm depth were also measured using the mini ion chamber array detector ICProfiler (Sun Nuclear Corporation, Melbourne, FL, United States). At the treatment plane, PDD curves and cross beam profiles at 1 cm depth of individual beam were measured by a plane‐parallel ion chamber inserted in a solid phantom.

The entire treatment was delivered to a cylindrical acrylic phantom with 30 cm diameter, an EDR film was sandwiched in the middle of the phantom under the central axis plane, and the dose distribution was analyzed to determine composite PDD, body factor and x‐ray contamination. Furthermore, the cylindrical phantom was replaced by a Rando phantom, OSLDs and films were placed on different locations (thigh, head, neck, mid‐mediastinum, CAX, upper thigh) to calculate the average level of x‐ray contamination and body factor that will be used clinically for MU calculation.

### 2.B Monte Carlo simulation

Several previous studies show that MC‐based dose calculations are sensitive to details of the source as well as the geometry of the linac head.^15^ Since the electron beam is normally shaped by the jaws and applicators, field size larger than 25 × 25 cm^2^ is rarely used in the clinic for standard electron therapy. Only few MC studies have covered the MC simulation of large electron fields.^15^, ^16^ In order to investigate the reliability of the simulation, we also used two independent MC codes, EGSnrc and VirtuaLinac, to simulate the Linac head.

#### 2.B.1 Original phase space files

The TrueBeam phase space files of 6 MeV electron beam, which were originally recorded at a plane just above the movable jaw at 26.7 cm away from the target and 73.3 cm from the isocenter using the Geant4 (version 10.0. patch1) code, were provided by the vendor in International Atomic Energy Agency (IAEA) compatible format.^17^ To avoid recycling the particles during the end‐user simulation, we first concatenated 20 phase space files to generate a source file containing the type (electron, photon, and positron), energy, positions, and direction cosines of more than 7.5 × 10^8^ pseudo‐particles in the region of interest.

#### 2.B.2 EGSnrc

The simulation was performed primarily within the EGSnrc environment,^11^ which has been accepted as the gold standard in the radiotherapy field. Under the EGS code, two packages were primarily used: BEAMnrc^18^ was aimed for modeling components in the Linac head as well as the in‐house built scattering filter that was used in TSI lay‐down technique;, and DOSXYZnrc^19^ used to calculate the dose deposition in the voxelized phantoms at different SSDs. Dose per primary particle as well as statistical uncertainty for each voxel, was scored in the 3ddose file format. Parameters and algorithms of particle transportation were chosen based on the recommendation of previous publications^11^, ^13^, ^17^ to balance the accuracy and efficiency of the simulation. Electron global cutoff energy (ECUT) and photon global cutoff energy (PCUT) were set to 0.521 and 0.01 MeV. During the simulation of Linac head in BEAMnrc, the electron range rejection variance reduction technique was turned on with an ESAVE GLOBAL of 1 MeV. Material and geometry of the downstream components were initially obtained from the TrueBeam Monte Carlo package (version 1.1, www.MyVarian.com).

From the origin plane to the isocenter (SSD = 100 cm), BEAMnrc^18^ was used with designated component modules (CM). Since the phase space file was field‐independent, secondary collimator of X and, Y jaws was modeled in BEAMnrc using JAWs CM to set the field sizes as 10 × 10, 20 × 20, 30 × 30, 30 × 40, 36 × 36, and 40 × 40 cm^2^ at isocenter. The photon MLC located under the X jaws was modeled by PYRAMIDS CM as a tungsten square ring with a 40 × 40 cm^2^ opening. For lay‐down technique, the scattering filter built by SLAB and Flatfilter CM was put right downstream of the accessory tray (material properties of copper and polycarbonate were manually defined in the Pegs4 file); for stand‐up technique, the filter was removed and other parameters stayed the same. Two scoring planes were defined during the simulation: Phase space files generated at exit window (58 cm from the target) were used for beam characteristic analysis, and phase space files generated at isocenter (100 cm from the target) were used as the source to the downstream simulation. A 60 × 60 × 10 cm^3^ water phantom with a voxel size of 1.0 × 1.0 × 0.1 cm^3^ was simulated in DOSXYZnrc for both open field (labeled as Open Field, or OPEN in the following plots) and filtered field (labeled as Cu Field, or Cu in plots) with different collimator settings (10 × 10, 20 × 20, 30 × 30, 36 × 36, and 40 × 40 cm^2^) at SSD of 100 cm.

To simulate AP_G60/300 fields for lay‐down technique, the phase space file recorded at 100 cm SSD was rotated by 60° at isocenter. A 200 × 100 × 10 cm^3^ water phantom with voxel size 2.0 × 2.0 × 0.2 cm^3^ was simulated at SSD = 195 cm in DOSXYZnrc. The rest of the region was modeled with air. For oblique fields, the model was simplified by positioning the phantom right underneath the linac head, the equivalent SSD was extended to 305 cm and the collimator angle was set to 90°. Dose profiles along X (patient longitudinal direction, superior to inferior), Y (patient lateral direction, left to right) directions and percent depth doses (PDDs) were calculated and compared.

To simulate and visualize the effects of all beams combined, we also created a cylindrical water phantom in the DOSXYZnrc with 190 cm length, 30 cm diameter and 0.1 × 0.1 × 2.0 cm^3^ voxel size (2.0 cm resolution is in the cranial–caudal direction), and position it at the same position as the actual treatment geometry. The outer space was filled with air. Each field was simulated separately, dose matrixes were rotated and combined with the weighting from those determined from commissioning. In addition, cylindrical phantoms with different diameters of 20, 25, 30, 35, and 40 cm were also simulated to evaluate the effect of varying patient sizes, along with different SSDs and SADs.

#### 2.B.2 VirtuaLinac

VirtuaLinac is a cloud‐based MC package developed by Varian and available for research purposes.^20^ It is operated on the Amazon Web Service (AWS) computation platform by requesting a spot to establish an instance. Phase space files were first uploaded, as well as the input xml file to control the gantry rotation and dose delivery. Phase space files with different collimator settings are generated at isocenter and compared with the phase space files generated from EGSnrc. A 60 × 60 × 10 cm^3^ water phantom with a voxel size of 1.0 × 1.0 × 0.1 cm^3^ was simulated at SSD = 100 cm. Although the newest version (1.4.9) of VirtuaLinac allows end‐users to add customized material of the Linac head, it is not verified yet; therefore, the results of the copper filter were not included in this paper.

To simulate TSI stand‐up technique, field size was set at 36 × 36 cm^2^ with gantry angles of 270 ± 19°, and a 200 × 100 × 10 cm^3^ water phantom with voxel size 2.0 × 2.0 × 0.2 cm^3^ was simulated at SSD = 300 cm. After the simulation, the dose matrix was downloaded and analyzed in Matlab (MathWorks, Inc., Natick, MA, United States).

### 2.C Comparison metrics

To validate the results of MC simulations, several key dosimetric indices were computed and compared with the measurement from the commissioning. For the dosimetry of open fields (profile at d_max_, PDD and absolute dose) results of EGS and Virtualinac were both calculated; results from only EGS are provided when the copper filter was included in the simulation, that is, the lay‐down technique. Unless explicitly stated, the results were from the EGSnrc code.

#### 2.C.1 Phase space file analysis

To study the effect of the Cu filter on beam characteristics, we analyzed the mean energy distribution and angular distribution of phase space files (with/without Cu filter included in the model) generated at SSD = 57.5 cm (exit window of the Linac head).

#### 2.C.2 Percentage depth dose and lateral profile of the individual field

Dose matrix post‐processing was performed by an in‐house built Matlab code, PDDs were calculated by averaging the neighboring four voxels at the same depth and then normalized to the maximum dose. Measured percentage depth ionizations (PDIs) in the water phantom were converted to PDDs by multiplying the stopping power ratio based on the methods reported in Supplement to the recommendation of Task Group 25.^21^ For PDDs comparison, parameters including percentage surface dose, depth of maximum dose (d_max_), depth of the 80% dose (R_80_), depth of 50% dose (R_50_), and x‐ray contamination at 10 cm depth were also compared.

Simulated lateral profiles at 1 cm depth were calculated by averaging the voxels in two neighboring depths and normalized to the value at the central axis. It was further smoothed by a median filter to preserve the field edges. Profiles at the same depth are also measured using the mini ion chamber array detector IC Profiler (Sun Nuclear Corporation, Melbourne, FL, USA). Dose difference between simulation and measurement was calculated.

#### 2.C.3 Output factor

The absolute dosimetry calibration was measured with a plane‐parallel ion chamber in a water phantom at the reference condition (36 × 36 cm^2^, d = 1.5 cm, no applicator) at the SSD of 100 cm. Relative output factors of individual field, defined as the ratio of dosimeter reading at treatment SSD to the reading at reference condition, were measured at the surface and 1 cm depth of the solid water phantom at treatment SSD, and then converted to the absolute dose per MU used for MU calculation. In the simulation, the dose was calibrated at the same reference condition, and relative output at treatment SSD was calculated following the same rule. Relative differences in the output factors between MC simulation and measurement were calculated.

#### 2.C.4 Percentage depth dose of composite fields

The dose matrixes of the individual field in the cylindrical phantom were first smoothed by averaging with neighboring voxels to improve the precision and reduce the noise, and then normalized by the maximum dose along CAX of AP field. Dose distribution in transverse planes of the cylindrical phantom at different off axis distances along the sup‐inf direction (off‐axis distance (OAD) = 0, 40, 80 cm) were also presented in the results section. Depth dose profiles from surface to the center were also computed at every 15°, composite PDDs were calculated by average the depth dose profiles at these 24 angles, which represents the mean penetration depth profile of the electron beam. For the measurement, an EBT3 film was placed inside the cylindrical phantom under the CAX of AP field, and further analyzed by an in‐house developed Matlab code.

To evaluate the dose variations in the phantom, the dose profile was extracted along the diameters of the cylinder at every 30° for further analysis at OAD = 0, 40, and 80 cm. Four representative axes were chosen: (a) the CAX of AP beam, or PDD0, (b) the CAX of oblique beam, or PDD60, (c) the middle between AP and one oblique beams, PDD30, and (d) the middle of two oblique beams, PDD90. Each axis was different from others due to the different setup of AP and OB beam. For stand‐up technique, two PDDs at axes are chosen: the region that is directly irradiated (PDD0) and the middle of two beams (PDD30).

#### 2.C.5 X‐ray contamination and body factor

Body factor (B‐factor) is defined as the ratio of the dose of the surface voxel at CAX from all beam pairs combined and a single beam pair. X‐ray contamination of the whole treatment is defined by the ratio of the dose at the center voxel of the central slice and the dose at the surface of the cylindrical phantom. Both values can be obtained from the measurements with OSLDs placed on the surface of the cylindrical phantom and at the center of the phantom.

#### 2.C.6 Investigation on the effect of patient size

In order to study the dosimetry of TSI technique as a function of different patient sizes, we computed the dose distribution in the cylindrical phantoms with diameters of 20, 25, 30, 35, and 40 cm. Gantry angle, phantom location on the floor, and MU prescription were the same with the original setup during the commissioning; however, SSDs and relative incident angle to the phantom may change. The quantitative analysis focused on three main parameters that may potentially vary with the phantom size: absolute dose at d_max_, which affects the actual dose delivery on the patient; B‐factor, which represents the cumulative effects of all fields; x‐ray contamination, which indicates the potential hazard to the inner organs.

## 3 RESULTS

### 3.A Linac head modeling results

For each step, 5 × 10^8^ histories were simulated. The statistical uncertainty of the simulated dose was 0.7% at d_max_, and gradually increased to 1.5% at fall‐off region. The uncertainty rose to 5–15% in the low dose bremsstrahlung region.

**Fig. 2:**
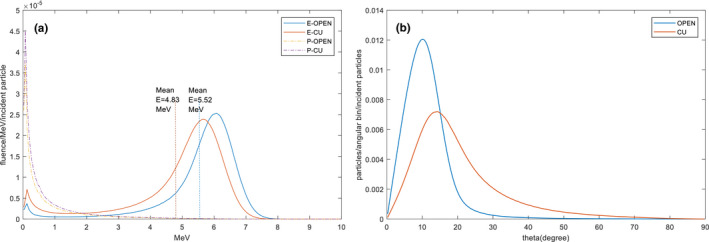
Effect of the copper filter on beam characteristics (a) energy spectrum of electron and photon (E: electron, P: photon) for the beam with/without copper filter (OPEN: without filter, CU: with filter mounted) at SSD = 57.5 cm (exit of the Linac head); (b) angular distribution of electrons for the beam with/without copper filter (OPEN: without filter, CU: with filter mounted).

At the Linac exit window, around 4.2 × 10^8^ pseudo‐particles, including photons, electrons, and positrons, were recorded in the phase space files. Figure 2(a) shows the energy spectrum of electrons and photons (number of positrons are <10^−5^ of the total particles and therefore was excluded in the analysis) and Fig. 2(b) shows the angular distributions of the incident electrons, respectively, indicating the influence of the filter to the incident electron beam in the following three aspects: (a) The mean energy of the electrons decreased by approximately 0.7 MeV; (b) The mean angular spectrum of the electrons were broadened by 9.1°; and (c) secondary photons increased by 24%, which might contribute to the increased photon contamination.

### 3.B Results in the water phantom at SSD of 100 cm

**Fig. 3:**
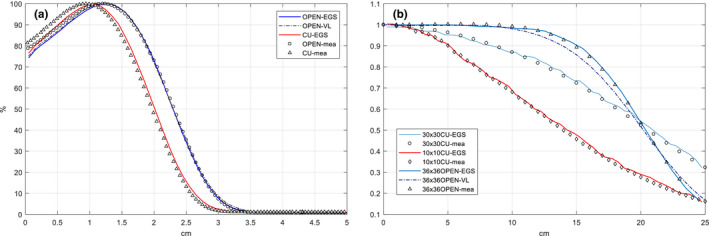
(a) Percentage depth doses comparison between MC simulation (OPEN: without filter, CU: with filter mounted) and measurement for a 36 x 36 cm^2^ field at SSD = 100 cm; (b) lateral profiles at 1 cm depth and SSD = 100 cm for MC simulation (solid lines) and measurement (markers), doses were normalized to the central axis values.

The measured and simulated PDDs for a 36 × 36 cm^2^ field at SSD = 100 cm are shown in Fig. 3(a): for open field PDDs, an agreement with 2%/1 mm criteria was achieved for all points excluding few surface voxels, EGSnrc(EGS) and Virtualinac(VL) agreed within 1%/1 mm; for Cu field PDDs, a larger deviation up to 4.5% was observed in the first few voxels of build‐up region, but the agreements were still within 2%/1 mm in the fall‐off region. Parameters of d_max_, R_80_, R_50,_ and contamination at 5 cm depth are listed in Table 2 and the differences between simulation and measurement were all <1 mm. By comparing PDD curves between open field and Cu field, we can observe that the copper filter caused the percentage surface dose increased 4%, d_max_, R_80_, and R_50_ shifted approximately 2 mm to the surface, and x‐ray contamination increased dramatically for the filtered field. In addition, lateral profiles at 1 cm depth of different collimator settings (10 × 10, 20 × 20, 30 × 30, 36 × 36, and 40 × 40 cm^2^ at SSD = 100 cm) were validated for both open field and filtered field;, several representative profiles with collimator setting that would be used for the TSI treatment are shown in Fig. 3(b), and the differences between simulation and measurement were within 3%. At SSD = 100 cm, full width half maximum (FWHM) of the filtered field increased by 44% in average with the same collimator setting, indicting a significant broadening effect of the filter.

**Table 2:** Comparison of open field and CU field between simulation and measurement.

PDD curves	Metrics	Simulation	Measurement	Difference (sim‐mea)
Open field (EGS/VL)	Surface dose (%)	(EGS:74.75; VL:74.25)	77.50	(2.75;3.25)
	d_max_ (cm)	(EGS:1.25; VL:1.25)	1.20	(−0.05; −0.05)
	R_80_ (cm)	(EGS:1.91; VL:1.88)	1.88	(−0.03; 0)
	R_50_ (cm)	(EGS:2.27; VL:2.27)	2.32	(0.05; 0.05)
	Contamination (%)	(EGS:0.58; VL:0.61)	0.80	(0.22; 0.19)
CU field (EGS)	Surface dose (%)	77.25	81.50	4.5
	d_max_ (cm)	1.05	0.98	−0.07
	R_80_ (cm)	1.62	1.58	−0.04
	R_50_ (cm)	1.97	1.90	−0.07
	Contamination (%)	0.92	1.2	0.28

### 3.C Results in the water phantom at extended SSDs for TSI

**Fig. 4:**
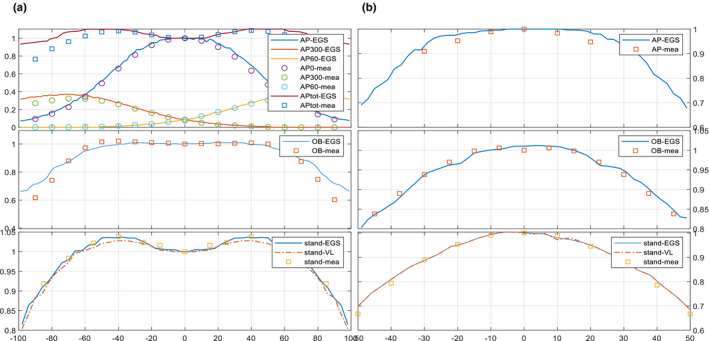
(a) Longitudinal (from patient superior to inferior) profiles comparison at 1 cm depth of lay‐down technique (AP_G0, AP_G60, AP_G300, OB fields) and stand‐up technique (stand) between simulation (EGS: solid line; VL: dashed line) and measurement (square mark), doses were normalized to the central axis values; (b) lateral (from patient left to right) profiles comparison at 1 cm depth of lay‐down technique (AP_G0, AP_G60, AP_G300, OB fields) and stand‐up technique between simulation (EGS: solid line; VL: dashed line) and measurement (square mark), doses were normalized to the central axis.

Figure 4 shows the dose profile (longitudinal: patient superior to inferior; lateral: patient left to right) comparisons at 1 cm depth at different treatment plane (AP: SSD = 195 cm; Oblique: SSD = 305 cm; Dual beam: SSD = 300 cm), doses were all normalized to the value at the central axis.

Deviation between simulation and measurement increased slightly with extended SSD and OAD. A general agreement within 5% was achieved.

**Fig. 5:**
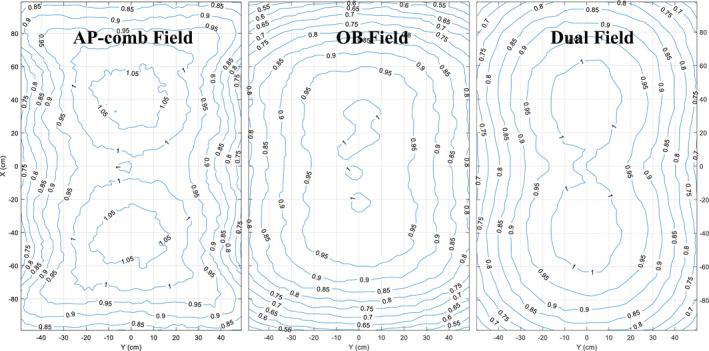
Planar dose distribution (MC‐simulated) at 1 cm depth for AP combined field, OB field in lay‐down technique and dual fields in stand‐up technique, doses were normalized to the values at central axis.

Planar dose distributions (MC‐simulated) of the fields at 1 cm depth in the phantom are shown in Fig. 5; doses in each voxel were normalized to the average dose at the central axis. Within ±10% difference from the prescription dose at the central axis, a flat central field of 180 × 80 cm^2^ was obtained for AP fields by an optimal weighting of 1:0.22:1 of AP_G60, AP_G0, and AP_G300. For oblique fields, because a single beam was delivered, a smaller field of 135 × 70 cm^2^ within ±10% difference was obtained. For the dual field used in stand‐up technique, the effective field size was 180 × 60 cm^2^. This is an example where the MC can be useful. Not only can it provide the dose values at certain points that can be verified by measurement, but also simulate the dose distribution of the whole area or volume for further analysis and decision making.

Table 3 shows the output factor at the central axis of the beam from both simulation and measurement, statistical uncertainties were included in the output from the simulation. For AP_G60 and AP_G300 fields, the output was determined by taking the average value of the individual measurement of each field. While both doses at depths of 0 cm (surface) and d_max_ of 1 cm were measured, the surface output was used for final MU determination since the prescription point locates at the surface region.

**Table 3:** Output obtained from both measurement and simulation, the output was calibrated at the reference condition (SSD = 100 cm, depth = 1.5 cm, Field size = 36 × 36 cm^2^, open field), uncertainty of the simulated value was included in the table.

Field name	Gantry (˚)	Depth (cm)	Output_meas_ (cGy/MU)	Output_sim_ (cGy/MU)	Difference (%)
Open field (iso)	0	1.5	1.0000	1.0000 ± 0.0792	/
Filtered field (iso)	0	1.5	0.4927	0.4971 + 0.0356	1.0
Vertex (AP & PA)	0	0	0.0830	0.0797 ± 0.0008	‐3.1
	60/300	0	0.0078	0.0081 ± 0.0004	3.8
	0	1	0.0983	0.0996 ± 0.0012	1.4
	60/300	1	0.0076	0.0079 ± 0.0004	3.9
Oblique	300	0	0.0292	0.0285 ± 0.0003	‐2.4
	300	1	0.0336	0.0336 ± 0.0004	0.1
Stand‐up (dual fields)	19/‐19	0	0.0724	EGS:0.0707 ± 0.0007 VL: 0.709 ± 0.0008	EGS:‐2.3 VL:‐2.1

### 3.D Composite dose in cylindrical phantom at extended SSDs for TS

**Fig. 6:**
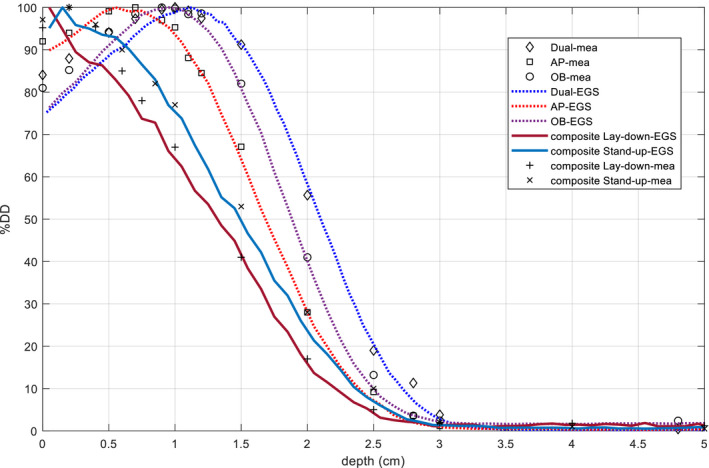
Comparison of percentage depth dose curves (from MC simulation) at center plane of the phantom for stand‐up technique and lay‐down techniques, composite PDDs were calculated by averaging PDDs from different angles with 15 degrees increments in the cylindrical phantom.

In the cylindrical phantom, simulated PDDs of individual field and composite PDDs are shown in Fig. 6; comparison of d_max_, R_80_, and surface dose between simulation and measurement are listed in Table 4 and differences are all within 2 mm. We found that d_max_ of both composite PDDs (composite lay‐down, composite stand‐up) shifted toward the surface from the single direction, and the difference of R_80_ was within 1 cm, meeting the dosimetric recommendation of TG‐30.

**Table 4:** Comparison of d_max_, R_80_ and surface dose of composite PDD and PDDs of individual field between simulation and measurement.

PDD		Metrics	Measurement	Simulation	Difference (sim‐mea)
Lay‐down	AP beam	d_max_ (cm)	0.67	0.58	−0.09
		R_80_ (cm)	1.40	1.25	−0.15
	OB beam	d_max_ (cm)	0.86	1.02	0.16
		R_80_ (cm)	1.40	1.55	0.15
	Composite PDD	Surface (%)	95.20	99.50	4.30
		d_max_ (cm)	0.15	0.05	−0.10
		R_80_ (cm)	0.75	0.75	0
Stand‐up	Dual beam	d_max_ (cm)	1.00	(EGS:1.05; VL: 0.95)	(0.05; −0.05)
		R_80_ (cm)	1.59	(EGS:1.7; VL: 1.65)	(0.11; 0.06)
	Composite PDD	Surface (%)	97.11	95.20	−1.91
		d_max_ (cm)	0.15	(EGS: 0.20; VL: 0.15)	(0.04; 0)
		R_80_ (cm)	0.92	(EGS:0.85; VL: 0.80)	(−0.07; −0.12)

**Fig. 7:**
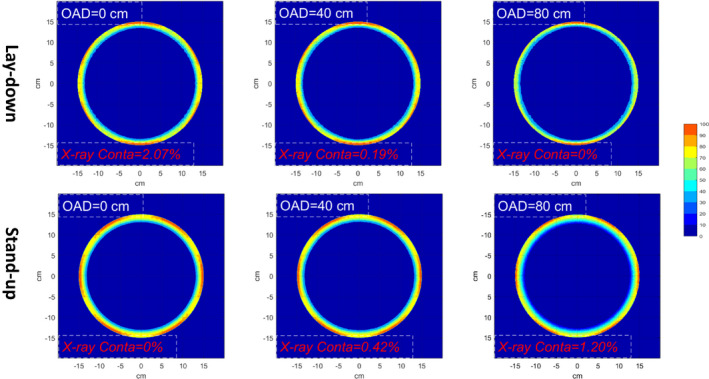
Dose distributions (MC simulated) of the lay‐down and stand‐up technique in the cylindrical phantom at different off‐axis distances (OAD = 0, 40, 80 cm). Dose in each voxel was normalized to the surface dose at CAX.

**Fig. 8:**
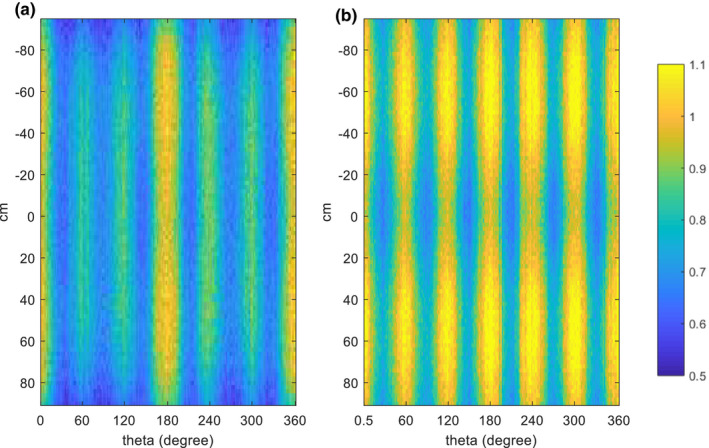
Dose map (MC simulated) at d_max_ = 0.15 cm in the cylindrical phantom, dose in each voxel is normalized to the surface dose at beam central axis. (a) Lay‐down technique; (b) stand‐up technique.

Dose distributions (MC‐simulated) from the complete treatment on the transverse plane of the cylindrical phantom were shown in Fig. 7. In addition, by unfolding the cylindrical phantom at d_max_ = 0.15 cm, dose maps for both stand‐up technique and lay‐down technique were shown in Fig. 8. The results showed that the surface region of the phantom have been completely covered by a 60% isodose curve, doses of regions that were not irradiated directly by the incident beam were slightly lower compared to the directly irradiated regions, but still satisfying the recommendation of TG‐30. For the lay‐down technique, x‐ray contamination at umbilicus level was 2.2% in the simulation, compared to 2.1% from the measurement, and gradually dropped to 0.2% at 40 cm off the central location; For the stand‐up technique, x‐ray contamination at umbilicus level was negligible, compared to <0.01% from the measurement, gradually increased to 0.42% at 40 cm off‐axis‐distance and 1.20% at 80 cm off‐axis‐distance. Body factor of the stand‐up technique and lay‐down technique in the simulation were 3.24, 3.54 (AP), and 3.51 (OB), which agreed with 3.10, 3.45 (AP), and 3.34 (OB) obtained from the measurement.

**Fig. 9:**
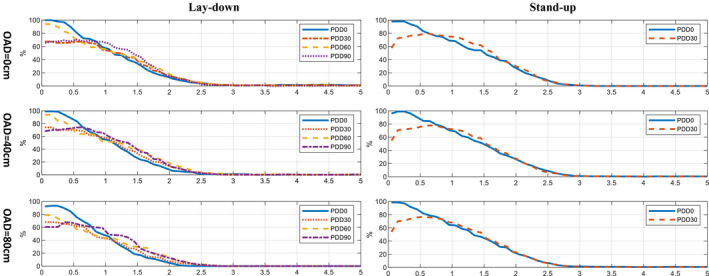
PDD curves (MC simulated) for different incident beam directions (0°, 30°, 60°, 90° counter‐clockwise from AP direction) at different off‐axis‐distance (0, 40, and 80 cm). Due to the symmetry of the profiles in the cylindrical phantom, only half of the curves are shown. Doses are normalized to the dose at the prescription point.

Figure 9 compared the PDDs at the center of direct irradiated region and the superimposed region at OAD of 0, 40, and 80 cm. For lay‐down technique, the surface dose at the superimposed regions (PDD30 and PDD90) dropped to 60–75% of the prescription dose, and R_50_ of PDD30 and PDD90 were slightly larger than the CAX of the irradiated regions (PDD0, PDD60). In addition, while the surface dose of four curves decreased as the OAD increased, the R_50_ also dropped slightly (3–4 mm), showing that the beam was less penetrating at the field edge, this was caused by the higher mean energy and larger angular deflection of the incident electrons.

**Fig. 10:**
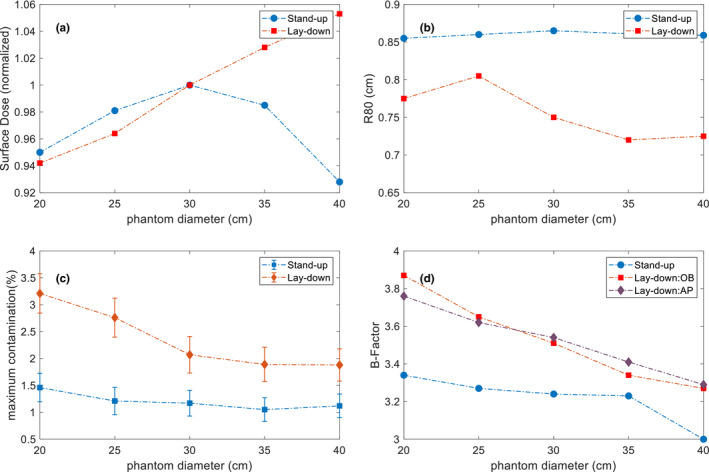
(a) MC‐simulated Surface dose (normalized to the dose in the cylindrical phantom with 30 cm diameter) in the cylindrical phantoms with 20, 25, 30, 35, and 40 cm diameter; (b) MC‐simulated R_80_ of the composite PDDs in the cylindrical phantoms with 20, 25, 30, 35, and 40 cm diameter; (c) MC‐simulated maximum x‐ray contamination (%) along the axis of the cylindrical phantoms with 20, 25, 30, 35, and 40 cm diameter; (d) MC‐simulated body factor calculated in the cylindrical phantoms with 20, 25, 30, 35, and 40 cm diameter.

With the same MU prescription used for different phantom size, general uniform dose distributions were achieved in all the phantoms simulated with different diameters. For the lay‐down technique, average dose at the surface [Fig. 10(a)] varied within 10%, cylindrical phantom of 20 and 40 cm diameters shows the surface dose of 94% and 105%, compared to 30 cm diameter. An approximate 3% increase on the surface dose was observed as phantom diameter increased every 5 cm. However, such a phenomenon did not appear on surface doses of the stand‐up technique. R_80_ [Fig. 10(b)] of the composite PDDs varied within 0.1 cm among different phantoms. X‐ray contamination [Fig. 10(c)] and body factor [Fig. 10(d)] shows an approximately linear decrease with increasing phantom sizes.

## 4 DISCUSSION

### 4.A Error analysis

Although both MC codes were used successfully to obtain an acceptable match to measured electron dose without using the applicator, Virtualinac was found to be slightly more accurate for the large field electron dose calculation; this is likely due to a well‐defined internal geometry of the Truebeam Linac head. Without accurate knowledge of the detailed information on the linac head, the user of EGSnrc cannot further optimize the current model to obtain higher accuracy. However, user codes in EGSnrc include precoded components modules, which allow end‐user to define customized geometry such as the copper filter used in TSI lay‐down technique. This feature makes it preferable for simulating nonstandard treatment techniques.

Measurements at the treatment plane can be difficult to set up as well, errors can likely occur due to the variation of SSD, OAD, and angular position. Other uncertainties that result in the difference between simulation and measurement is the room scatter effect, as our MC simulation model cannot account for every detailed geometry of the treatment room. Previous study has shown that walls and ceilings could contribute up to 20% of the doses at different off‐axis distances,^14^ which can also be identified in the increased difference of the dose profile between simulation and measurement at large OADs (OAD > 70 cm).

Previous study^17^ has validated the electron beam dose calculations for electron beam energies of 6–20 MeV using phase space files for open and collimated field sizes from 3 × 3 to 25 × 25 cm^2^ with measurements on Varian TrueBeam linacs. However, with the applicator removed and the jaws wide open, increasing scattering electrons contributes significantly to the dose at the periphery region. Therefore, the large electron field may prove a superior beam for extracting parameter values for these models. By comparing PDDs, and relative dose profiles between MC simulation and measurement, we validated the beam output for the large electron field (30 × 30, 36 × 36, and 40 × 40 cm^2^) using the phase space files of 6 MeV electron beam, which supplemented the previous work of our group.^17^, ^22^ Furthermore, we validated the dosimetry at extended SSDs. From a broad perspective, feasibility of MC simulation in nontraditional treatment was explored, which proved that MC simulation can be a promising tool to reduce the time and physics resources at the facility when a new technique is commissioned.

### 4.B Comparison between TSI stand‐up and lay‐down technique

We noticed that the effective field size and incident energy were not the same for vertex fields and oblique fields. While vertex beams can provide an equivalent field size (even better) compared to stand‐up technique, a single field for oblique beam (135 × 70 cm^2^) can barely encompass the largest patient. Therefore, the lay‐down technique may not achieve the same degree of coverage as the stand‐up technique.

While d_max_ of composite PDDs for both techniques are in the same depth, R_80_ of the lay‐down technique suggests that the beam is less penetrating compared with stand‐up technique. This can be either advantageous or disadvantageous, depending on the specific disease location. Therefore, such difference should be kept in mind when a specific technique is chosen for the patient treatment.

Doses at the central axis of the cylindrical phantom suggest an increased value of x‐ray contamination for lay‐down technique. Maximum xray contamination reached 2%, slightly higher than the 1% level of stand‐up technique. This is primarily due to the effect of the scattering filter: interaction between the electron beam and the high‐Z material degrades the electron energy, increases the angular distribution of the electron particles and produces secondary photons, resulting in a significant increase of the photon component in the electron. Reduced SSD for AP fields may also contribute to the increased x‐ray contamination, as electron dose falls off much faster as a function of SSD. In addition, the use of the gantry angle of 0 degree for AP and PA direction may contribute to this also, as the standup technique does not contain beam normal to the patient surface.

As a conclusion, with the similar dosimetric results validated by both measurement and MC simulation, lay‐down technique can be a good supplement in the TSI treatment, especially useful for those patients who cannot endure the standing position for the duration of treatment.

## 5 CONCLUSIONS

We developed a MC framework to simulate two methods for TSI. TSI stand‐up technique is easy to implement and most commonly used in the clinic. TSI lay‐down technique is implemented when the patient is unable to endure the stand‐up technique. MC simulation was used to examine and validate the dosimetry on water phantoms for the single field and combined fields. The results of our MC calculations were found to be in generally good agreement with the measurements, which provides secondary support in our commissioning procedure. To the best of our knowledge, this is the first time that a TSI lay‐down treatment is simulated in MC and compared with the standup technique. In addition to those measurable quantities, the MC simulation can provide further information such as the full dose distribution of the patient phantom, and the ability to investigate and optimize techniques such as different filter design, SSD, and field size variations.

## CONFLICT OF INTEREST

There is no conflict of interest for all authors.
